# A qualitative study of patients’ experiences of screening for psoriatic arthritis

**DOI:** 10.1111/bjd.21825

**Published:** 2022-09-02

**Authors:** Christine A. Silverthorne, Clive Bowen, Jane Lord, Neil McHugh, William Tillett, Emma Dures, Anya Lissina

**Affiliations:** ^1^ University of the West of England Bristol UK; ^2^ University of Bristol Academic Rheumatology Group Bristol UK; ^3^ University of Bath Bath UK; ^4^ on behalf of the PROMPT study group

dear editor, Psoriatic arthritis (PsA) is a chronic inflammatory arthritis which can cause pain, fatigue, swelling and stiffness in the joints, and can result in limited physical function and a high psychosocial burden.^1^ Patients with psoriasis are at greater risk of developing PsA than those without.^2^

There is no definitive test for PsA. A diagnosis is made by rheumatologists after referral from primary care based on the patient’s medical history, a physical examination, blood tests, a magnetic resonance imaging (MRI) scan and X‐rays. Delays can be due to patient‐related factors (e.g. reluctance to seek medical help) and clinician‐related factors (e.g. the lack of autoimmune diagnostic markers). Even a 6‐month delay from symptom onset can result in worse long‐term physical function.^3^

The PROMPT programme (National Institute for Health Research grant: RP‐PG‐1212‐20007) is investigating the clinical and cost benefits of early detection of PsA. The main study is a two‐arm parallel‐group cluster randomized controlled trial (RCT) of screening (known as TUDOR), using enhanced surveillance for PsA in primary care vs. standard care. Screening included an examination of participants’ skin, joints, hands, feet, scalp, physical tests (e.g. touching toes), height and weight measurement, blood tests, X‐rays, MRI scans and questionnaires. An important aspect of TUDOR is understanding whether screening would be acceptable to patients with psoriasis, some of whom will be diagnosed with PsA as a result and some of whom will not. The aim of this study was to understand the experience of screening from the perspective of participants with psoriasis recruited to the enhanced surveillance arm in the TUDOR RCT.

The study was approved by the Proportionate Review Sub‐Committee of the North East – Newcastle & North Tyneside 1 Ethics Committee (reference 16/NE/0393) and the Health and Applied Sciences Faculty Research Ethics Committee of the University of the West of England (reference: HAS.17.03.129). A qualitative design was used. Data were collected in one‐to‐one, semistructured, telephone interviews and analysed using framework analysis.^4^ Twenty‐four participants were recruited from two sites in the TUDOR RCT (Table 1). Three main themes represent the data.

**Table 1:** Participant data

Diagnosis	Male/female sex	Age (years)	Time with psoriasis (years)	Heard of PsA	Site	Interview date
PsA	Male	71	54	No	1	11 November 2019
PsA	Female	39	22	No	1	15 November 2019
PsA	Female	70	60	Yes	1	20 November 2019
OA	Female	58	6	No	1	22 November 2019
PsA	Male	40	30	No	1	25 November 2019
None	Female	59	15	No	1	2 December 2019
OA	Female	73	50	Yes	1	6 December 2019
PsA	Male	39	12	No	2	11 December 2019
None	Female	70	30	Yes	2	12 December 2019
PsA	Male	49	24	No	2	12 December 2019
PsA	Male	58	57	Yes	1	13 December 2019
None	Female	72	30	No	1	10 January 2020
None	Female	56	51	No	2	15 January 2020
OA	Female	66	38	Yes	1	15 January 2020
PsA	Male	43	22	No	1	24 January 2020
None	Male	62	43	Yes	2	2 March 2020
None	Female	40	24	No	1	3 March 2020
None	Female	56	38	No	2	5 March 2020
Crohn disease	Female	71	20	Yes	1	6 March 2020
PsA	Male	72	7	No	2	15 May 2020
None^a^	Female	56	38	Yes	2	15 May 2020
PsA	Female	61	55	Yes	2	18 May 2020
PsA	Male	55	20	Yes	2	18 May 2020
None	Female	35	18	Yes	2	21 May 2020

OA, osteoarthritis; PsA, psoriatic arthritis. ^a^Awaiting diagnosis at time of interview.

Theme 1 reports participants’ views on screening as part of healthcare. Participants described a range of feelings from apprehension and mild anxiety through to excitement and optimism. Overall, screening was a well‐conducted, positive and reassuring experience. Participants appreciated the thoroughness of the examination and the time to talk with specialists, including having the opportunity to talk about the impact of their condition on their mental health. This was the case both for participants who screened positive for PsA and for those who did not. Some felt that if it were not for the screening, they would still be experiencing pain and fatigue and living with undiagnosed PsA. For some participants screening resulted in other conditions being diagnosed (e.g. osteoarthritis, Crohn disease) and they were able to receive advice and help for these conditions.

Theme 2 reports participants’ thoughts on how screening enhanced a sense of control over their health. Participants who screened positive for PsA valued the help they were given in treating their condition, while those who screened negative gained an awareness of symptoms to watch out for and the need to seek advice quickly and initiate treatment if necessary. For some, diagnosis was a ‘lightbulb moment’ where the reason for their stiff, achy joints became clear. Participants referred to having made changes that were beneficial to their health following screening, for example making changes to diet and exercise, and adapting the way they did things and talked to others (including employers) about their PsA.

Theme 3 reports views on optimizing screening. Suggested improvements include using case studies, signposting to support groups and information provision. Participants mentioned barriers to attending screening, including location, parking and time of appointments. Other potential barriers included embarrassment (especially if clothing needed to be removed) and concerns about what to expect.

These findings showed that screening was acceptable to participants whether they were diagnosed with PsA or not. This supports a systematic review and thematic synthesis of qualitative studies which found that participants felt empowered when they understood the link between psoriasis and PsA.^5^ In addition to possible diagnosis, screening appointments provided an environment to support participants’ self‐management by communicating advice about the nature and treatment of PsA, addressing negative illness beliefs and unhelpful coping strategies that participants may have developed to deal with their psoriasis, and by encouraging participants’ ownership of their health condition.^6^, ^7^ This contrasts with participants’ experiences of being diagnosed with PsA in the current healthcare context, where those who had experienced disbelief and misdiagnoses could arrive in rheumatology already anxious, in pain and distrustful of healthcare providers.^8^

This qualitative study indicates that screening is acceptable and a potentially valuable method to increase the early detection of PsA in patients with psoriasis and improve their clinical outcomes.

## Author contributions

**Christine Silverthorne:** Data curation (lead); formal analysis (lead); investigation (lead); methodology (equal); project administration (lead); visualization (equal); writing – original draft (lead); writing – review and editing (equal). **Clive Bowen:** Validation (supporting); visualization (supporting); writing – review and editing (supporting). **Jane Lord:** Validation (supporting); visualization (supporting); writing – review and editing (supporting). **Neil McHugh:** Conceptualization (equal); funding acquisition (equal); visualization (equal); writing – review and editing (equal). **William Tillett:** Conceptualization (equal); validation (equal); visualization (equal); writing – review and editing (equal). **Emma Dures:** Conceptualization (equal); formal analysis (equal); methodology (equal); supervision (lead); visualization (equal); writing – review and editing (equal). **Anya Lissina:** Conceptualization (equal); funding acquisition (equal); resources (equal).

## Data Availability

The data that support the findings of this study are available from the corresponding author upon reasonable request." cd_value_code="text
